# A novel pyrazole derivative protects from ovariectomy-induced osteoporosis through the inhibition of NADPH oxidase

**DOI:** 10.1038/srep22389

**Published:** 2016-03-15

**Authors:** Jung Hee Joo, Jeong-Eun Huh, Jee Hyun Lee, Doo Ri Park, Yoonji Lee, Seul Gee Lee, Sun Choi, Hwa Jeong Lee, Seong-Won Song, Yongmi Jeong, Ja-Il Goo, Yongseok Choi, Hye Kyung Baek, Sun Shin Yi, Soo Jin Park, Ji Eun Lee, Sae Kwang Ku, Won Jae Lee, Kee-In Lee, Soo Young Lee, Yun Soo Bae

**Affiliations:** 1 Department of Life Science, Ewha Womans University, Seoul, Republic of Korea; 2 College of Pharmacy and Graduate School of Pharmaceutical Sciences, Ewha Womans University, Seoul 120-750, Korea; 3 Bioresearch Institute, Yooyoung Pharmaceuticals Co. Ltd., Seoul 152-719, Republic of Korea; 4 College of Life Sciences and Biotechnology, Korea University, Seoul 136-701, Korea; 5 Department of Biomedical Laboratory Science, College of Medical Sciences, Asan 31538, Soonchunhyang University; 6 Department of Anatomy and Histology, College of Oriental Medicine, and The Medical Research center for Globalization of Herbal Formulation, Daegu Haany University, Gyeongsan 712-715, Republic of Korea; 7 School of Biological Science, Seoul National University and National Creative Research Initiative Center for Symbiosystem, Seoul National University, Seoul 151-742, South Korea; 8 Green Chemistry Division, Korea Research Institute of Chemical Technology, P.O. Box 107, Yuseong, Taejon 305-600, Korea

## Abstract

Osteoclast cells (OCs) are differentiated from bone marrow-derived macrophages (BMMs) by activation of receptor activator of nuclear factor κB (NF-κB) ligand (RANKL). Activation of NADPH oxidase (Nox) isozymes is involved in RANKL-dependent OC differentiation, implicating Nox isozymes as therapeutic targets for treatment of osteoporosis. Here, we show that a novel pyrazole derivative, Ewha-18278 has high inhibitory potency on Nox isozymes. Blocking the activity of Nox with Ewha-18278 inhibited the responses of BMMs to RANKL, including reactive oxygen species (ROS) generation, activation of mitogen-activated protein (MAP) kinases and NF-κB, and OC differentiation. To evaluate the anti-osteoporotic function of Ewha-18278, the derivative was applied to estrogen-deficient ovariectomized (OVX) ddY mice. Oral administration of Ewha-18278 (10 mg/kg/daily, 4 weeks) into the mice recovered bone mineral density, trabecular bone volume, trabecular bone length, number and thickness, compared to control OVX ddY mice. Moreover, treatment of OVX ddY mice with Ewha-18278 increased bone strength by increasing cortical bone thickness. We provide that Ewha-18278 displayed Nox inhibition and blocked the RANKL-dependent cell signaling cascade leading to reduced differentiation of OCs. Our results implicate Ewha-18278 as a novel therapeutic agent for the treatment of osteoporosis.

Osteoporosis is a condition in which calcified bone density is decreased and the compact substance of bone is lost gradually, leading to broadening of the marrow cavity^1^,^2^. As osteoporosis progresses, bone becomes fragile and bone fractures may readily occur even with a small impact. Bone mass is affected by a variety of factors including genetics, nutrition, hormonal changes, physical exercise and lifestyle habits. Aging, insufficient exercise, being underweight, smoking, low-calcium dietary intake, menopause and ovariectomy are pathogenic causes of osteoporosis. Loss of bone mass by various pathogenic conditions is induced by bone remodeling^3^,^4^,^5^.

Bone remodeling involving removal and replacement of bone is coupled to maintain the balance of bone volume^6^,^7^,^8^. Two major cell types, osteoblasts (OBs), and OCs, are involved in bone remodeling. Bone marrow stromal cells differentiate into OBs that synthesize bone matrix and participate in bone formation, whereas OCs derived from hematopoietic stem cells are involved in bone resorption. Equilibrium between bone formation and bone resorption plays a critical role in the homeostasis of bone. Multinuclear OCs differentiate from a monocyte/macrophage lineage of hematopoietic progenitor cells through a multi-stage process of cell adhesion, proliferation, motility, cell-cell contact and terminal fusion for the formation of multinucleated giant cells. This process is mediated by binding of a receptor activator of NF-κB ligand (RANKL) to its receptor, RANK and is then transmitted through the activation of several signaling cascades. The activated signaling pathway includes NF-κB, extracellular signal-regulated kinase (ERK), c-Jun, N-terminal kinase (JNK) and p38 mitogen-activated protein kinase (MAPK) through tumor necrosis factor (TNF) receptor-associated factor 6 (TRAF6)^9^,^10^,^11^,^12^. Such a signaling event has a direct effect on the modulation of differentiation and action of OCs.

The generation of reactive oxygen species (ROS) is involved in OCs differentiation^13^. Pretreatment of bone marrow-derived macrophages (BMMs) with antioxidants such as N-acetyl cysteine (NAC) and ascorbic acid results in inhibition of ovariectomy (OVX)-induced bone loss, suggesting that ROS serve as second messengers in osteoclastogenesis^14^. Moreover, Nox1-mediated ROS production stimulates the RANKL-dependent cell signaling cascade, leading to OC differentiation^15^. Two reports suggested that RANKL stimulated Nox2 and Nox4 expression in macrophage cell line (RAW264.7) and BMMs. Macrophage cells from Nox2 knockout mice produced ROS and Nox2 knockout mice did not show bone abnormality^16^. However, Nox4 knockout mice displayed higher bone density and reduced numbers of OCs^17^. These results are consistent with the hypothesis that the generation of ROS in OCs is dependent on the activity of NADPH oxidase and directly connected with osteoclastogenesis.

In the present study, we hypothesized that an anti-osteoporosis agent may be developed by taking advantage of a molecular mechanism that inhibits the generation of ROS. Here we show that a novel pyrazole compound (Ewha-18278) reduced OC differentiation through Nox inhibition, and thus the compound may be useful in the treatment of osteoporosis.

## Results

### Identification of Nox inhibitor

**Figure 1:**
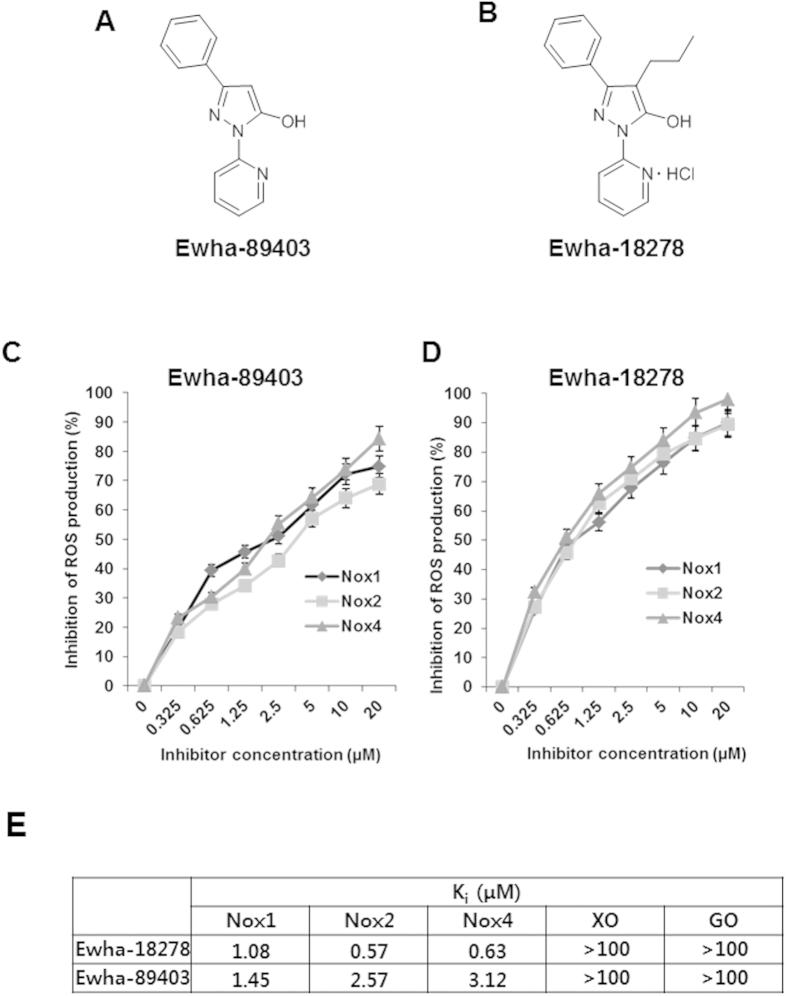
Chemical structure and Nox inhibition profile of Ewha-89403 and Ewha-18278. (**A**) Chemical structure of Ewha-89403. (**B**) Chemical structure of Ewha-18278. (**C**,**D**) Concentration-dependent Nox inhibition curves of Ewha-89403 (**C**) and Ewha-18278 (**D**). Drosophila membranes specifically overexpressing hNox1, hNox2, and hNox4 were subjected into ROS measurement with Diogene. (**E**) Ki value of Ewha-89403 and Ewha-18278 on hNox1, hNox2, hNox4, xanthine oxidase (XO) and glucose oxidase (GO).

Based on the notion that the differentiation of OCs is mediated by Nox1/2/4-dependent ROS generation, we have performed a high throughput enzyme inhibitor screening assay to identify chemical compounds having Nox1/2/4 inhibitory activity for the development of an anti-osteoporosis agent. Purified *Drosophila* membranes expressing human Nox1, Nox2, or Nox4 were used to monitor ROS production by determining the oxidation of lucigenin, evident as chemiluminescence, in the absence or presence of chemical compound^18^. A chemical library containing 31,000 compounds was screened to identify Nox inhibitors. We identified 3-phenyl-1-(pyridin-2-yl)-1H-5-hydroxypyrazol, designated Ewha-89403 as a potent inhibitor (Fig. 1A, Supplementary Fig. S1). A structure-activity relationship experiment with Ewha-89403 was conducted for a series of 304 compounds synthesized the peripheral modification of the parent molecule. This study revealed that the introduction of a propyl group to Ewha-89403 produced a novel chemical compound with enhanced physicochemical properties – Ewha-18278 [3-phenyl-1-(pyridin-2-yl)-4-propyl-1-5-hydroxypyrazol HCl] (Fig. 1B, Supplementary Fig. S2). *Ki* values for Nox isozymes of Ewha-18278 and Ewha-89403 were 0.57–1.08 μM and 1.45–3.56 μM, respectively. It indicates that both compounds showed good inhibitory activity on Nox1/2/4 isozymes (Fig. 1C–E). However, both compounds showed no inhibitory activities on xanthine oxidase and glucose oxidase (Fig. 1E). Moreover, we investigated whether Ewha-18278 can directly scavenge ROS *in vitro*. We added hydrogen peroxide in the presence or absence of Ewha-18278 and then measured changes in the hydrogen peroxide concentration. N-acetyl cysteine as a control ROS scavenger led to a decrease in hydrogen peroxide concentration, whereas Ewha-18278 had no effect. The result indicated that Ewha-18278 does not contain scavenging function of ROS (Supplementary Fig. S3A).

**Figure 2:**
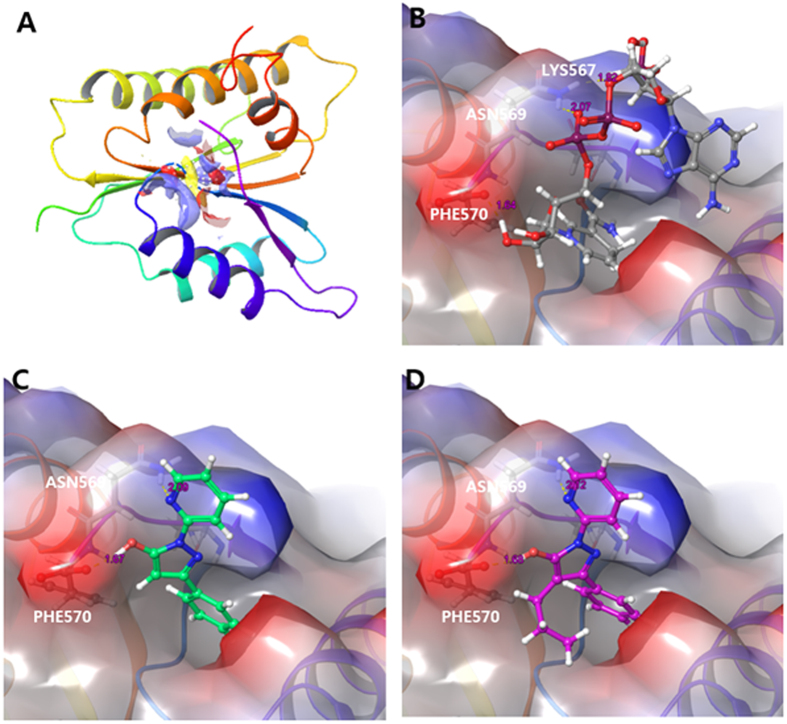
(**A**) Determination of docking site of crystal structure of NADPH binding domain of gp91^phox^ (PDB ID: 3A1F). (**B**) NADPH was bound the NADPH binding domain of gp91^phox^. (**C**) Ewha-89403 (Green) was bound the NADPH binding domain of gp91^phox^. (**D**) Ewha-18278 (purple) was bound the NADPH binding domain of gp91^phox^.

To evaluate binding site prediction of Ewha-89403 and Ewha-18278 with Nox isozyme, we used Sitemap with structure of gp91^phox^/Nox2 (Fig. 2A). The gp91^phox^/Nox2 structure bound with NAPDH, Ewha-89403, and Ewha-18278 were verified with Glide protein-protein docking application in Schrödinger suites. Glide generated ligand poses that have favorable interactions with the receptor employing a series of hierarchical filters. It begins with rough matching atom with receptor and generated possible ligand poses in the binding site based on Grid points. In the final stage, filtered ligand poses are scored with GlideScore scoring function and ranked. NADPH and compound Ewha-18278 and Ewha-89403 were shown on the 3D conformation (Fig. 2B–D). The phosphate group of NADPH was bound to NADPH binding site by forming a hydrogen bonds with ASN569, PHE570 and LYS567 of gp91^phox^/Nox2, (Fig. 2B). In common with NADPH, Ewha-89403 was bound to the NADPH binding site by forming two hydrogen bond interactions with PHE570 of gp91^phox^/Nox2 (Fig. 2C). Similarly, Ewha-18278 was bound to the NADPH binding site through hydrogen bond interaction with ASN569 and PHE570 (Fig. 2D). Based on the docking result, we predict that Ewha-18278 and Ewha-89403 are able to interrupt the interaction between NADPH and NADPH binding domain of gp91^phox^/Nox2 and other Nox isozymes. Ewha-18278 displayed a high oral availability compared to Ewha-89403 (Supplementary Table S1 and S2). The results indicate that Ewha-18278 has the physico-chemical property suitable for oral administration.

Because Nox2 has been well characterized for phagocytic function for host defense, we carried out additional experiments to assess whether the compound affects immune response such as phagocytosis of macrophages. The result indicated that the compound does not affect phagocytic function of BMMs (Supplementary Fig. S3B).

### Effect of Ewha-18278 on RANKL-induced OC differentiation

**Figure 3:**
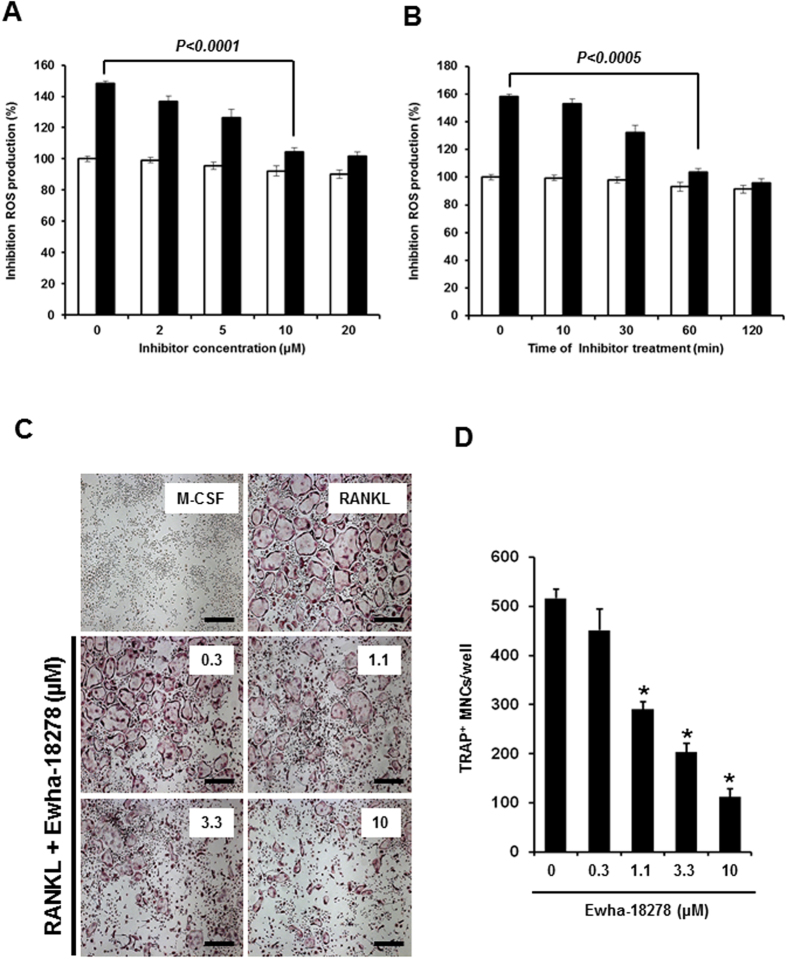
Ewha-18278 inhibits RANKL-dependent ROS generation and OC differentiation. Concentration (**A**) and time-dependent (**B**) Nox inhibition of Ewha-18278. BMMs were pretreated with indicated concentration of Ewha-18278 for 1 hr (**A**) or 10 μM Ewha-18278 for indicated times. After pretreated Ewha-18278, cells stimulated with RANKL (200 ng/ml) for 10 minutes. RANKL-induced ROS generation was monitored by confocal microscopic analysis of DCF fluorescence. Data represent three repeated experiments and are shown as mean ± SD (n = 3). (**C**) BMMs were treated with increasing concentrations of Ewha-18278 in the presence of M-CSF (30 ng/ml) and RANKL (100 ng/ml) and stained for TRAP activity. (**D**) TRAP^+^ MNCs containing more than 3 nuclei were counted. Data represent mean ± S.D., *P < 0.005. Scale bar, 500 μm.

Nox1, Nox2, and Nox4 have been suggested to be involved in RANKL-mediated OC differentiation. To evaluate the inhibitory role of Ewha-18278 in RANKL responses, the effect of Ewha-18278 on ROS generation was tested in BMMs. The addition of Ewha-18278 reduced RANKL-mediated ROS generation in dose- and time-dependent manners (Fig. 3A,B). A plateau of inhibition of RANKL-dependent ROS generation in BMMs was reached at 10 μM Ewha-18278 after 60 min. The result indicated that optimal inhibitory effect of Ewha-18278 on RANKL-mediated ROS generation in BMMs is obtained by treating for 60 min at 10 μM.

The fact that treatment of OC precursors with Ewha-18278 blocked RANKL-mediated ROS production indicating the potential value of Ehwa-18278 as an alternative means of inhibiting RANKL-induced OCs differentiation. To test this notion, the effect of Ewha-18278 on OCs differentiation was examined. Ewha-18278 treatment inhibited the formation of TRAP-positive OCs with high numbers (>3) of nuclei in a dose-dependent manner (Fig. 3C,D). Unlike its effect on OC differentiation, Ewha-18278 did not affect the fusion and bone resorption activity of OCs (Supplementary Fig. S4). In addition, we have investigated the effect of Ewha-18278 on cell death of OCs since ROS are known to regulate cell death in a variety of cell types. Treatment of Ewha-18278 had no effect on apoptosis of OCs in either the absence or presence of RANKL (Supplementary Fig. S5). Meanwhile, osteoblast (OB) differentiation from pre-OB cells was not affected by treatment of Ewha-18278 in the presence of osteogenesis induction media (Supplementary Fig. S6).

### Ewha-18278 suppresses RANKL-mediated cell signaling

**Figure 4:**
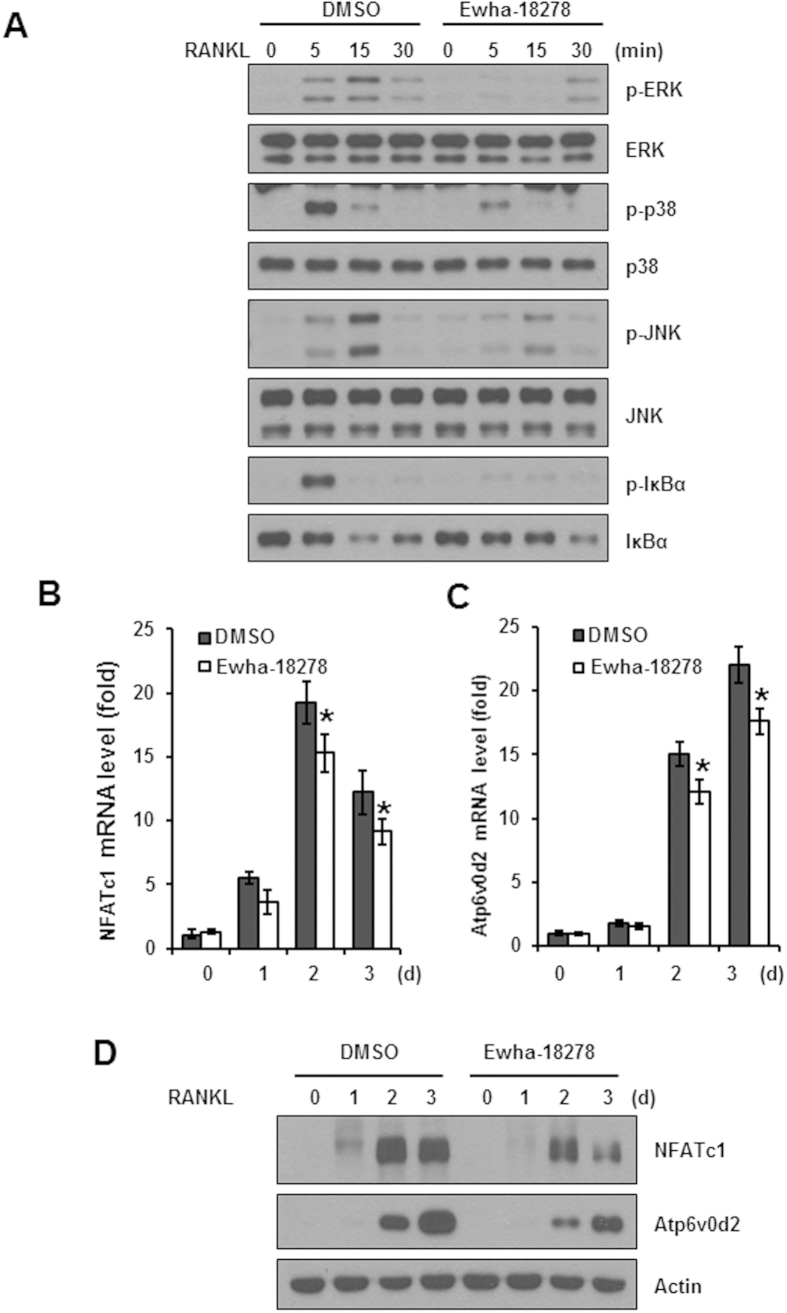
Effect of the Ewha-18278 on the RANKL-induced signaling pathways. (**A**) BMMs were pretreated with Ewha-18278 for 1 hour, the cells were stimulated with RANKL for the indicated times. The cell lysates were subjected to immunoblot analysis. RANKL signaling pathways assessed by phosphorylation of ERK, p38, JNK, and IκBα. Immunoblots were stripped and then reprobed with total ERK, p38, JNK, and IκBα. (**B**,**C**) BMMs were treated with Ewha-18278 in the presence of M-CSF and RANKL for the indicated periods. Total RNA was extracted from the cells and used in real-time PCR to quantify the mRNA levels of NFATc1 (**B**) and Atp6v0d2 (**C**) genes. The relative expression levels were normalized to the level of actin mRNA as an internal control gene. *P < 0.05. Data represent mean ± SD. (**D**) NFATc1 and Atp6v0d2 expression was determined by immunoblot analysis. Actin serves as a loading control.

Since RANKL-mediated ROS production serves to regulate RANKL-mediated cell signaling pathways, we examined whether Ewha-18278 affected RANKL-induced cellular signaling cascades. Treatment of BMMs with Ewha-18278 resulted in suppressed activation of MAPKs including JNK, p38 and ERK as well as phosphorylation of IκBα (Fig. 4A). Moreover, the expression levels of NFATc1 as an essential transcription factor for osteoclastogenesis as well as Atp6v0d2 as a well-known NFATc1 downstream target gene, were also decreased by the treatment of Ewha-18278 (Fig. 4B–D). Taken together, these results indicate that Ewha-18278 blocks RANKL responses including ROS generation, activation of MAPKs and NF-κB leading to suppressed OC differentiation.

### Effect of Nox inhibitor on OVX-induced osteoporosis

**Figure 5:**
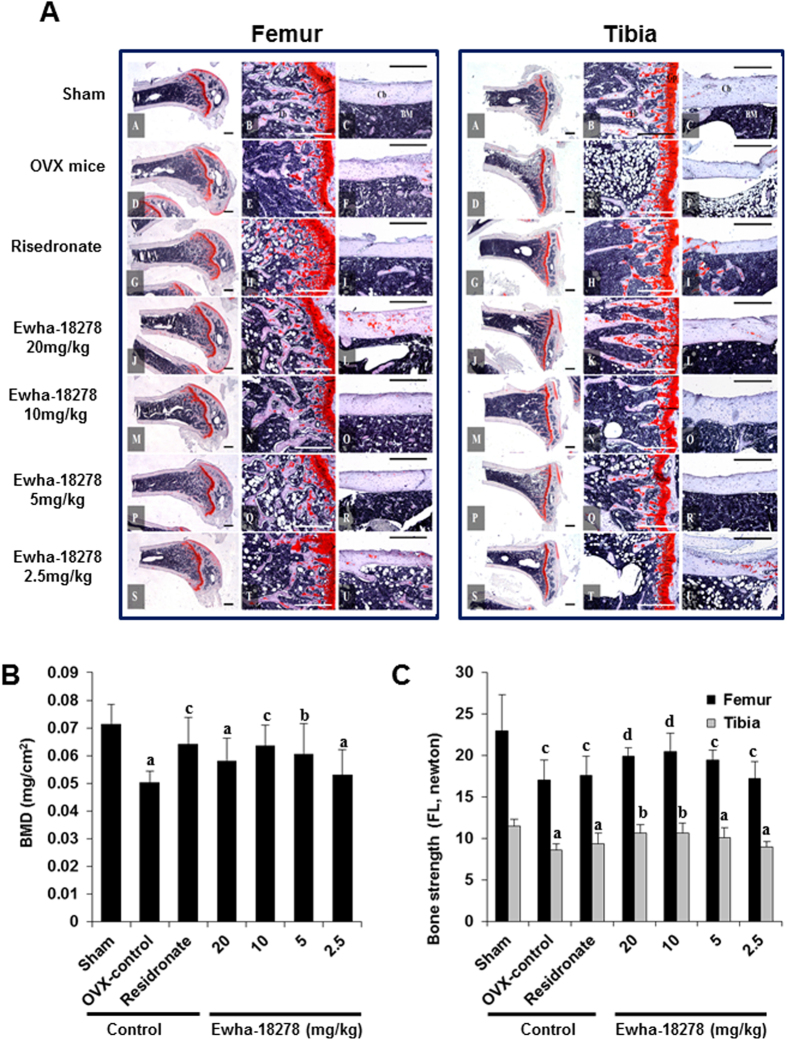
Representative histological profiles of the femur and tibia from OVX mice. (**A**) After 4 weeks after OVX surgery, eight mice per group were selected based on the body weights. Ewha-18278 was orally administered once a day for 28 days to OVX osteoporotic mice at 2.5, 5, 10 and 20 mg/kg levels in a volume of 10 ml/kg using 10% N, N-Dimethylacetamide (DMAC) +10% Tween 80 + 80% distilled water as vehicle. The histological profiles and histomorphometrical analyses of femur and tibia at sacrifice were conducted. The results of anti-osteoporotic effects of Ewha-18-278 were compared with risedronate sodium 2.5 mg/kg treated OVX mice in the present study. A–C = Sham control mice; D–F = OVX control mice; G–I = risedronate 2.5 mg/kg treated mice; J–L = Ewha-18278 20 mg/kg treated mice; M–O = Ewha-18278 10 mg/kg treated mice; P–R = Ewha-18278 5 mg/kg treated mice; S–U = Ewha-18278 2.5 mg/kg treated mice; Cb = cortical bone; Tb = trabecular bone; Bm = bone marrow; Gp = growth plate. All Safranin O stain and Scale bars = 480 μm. (**B**) Right Femur BMD in OVX Mice. Values are expressed mean ± S.D. of eight mice. Risedronate sodium was administered at 2.5 mg/kg levels. Ewha-18278 = test material. OVX = ovariectomy, BMD = bone mineral density, ^a^p < 0.01 and ^b^p < 0.05 as compared with sham control by LSD test. ^c^p < 0.01 and ^d^p < 0.05 as compared with OVX control by LSD test. (**C**) Right femur and tibia bone strength (FL, failure loading) in OVX mice. Values are expressed mean ± S.D. of eight mice. Risedronate sodium was administered at 2.5 mg/kg levels. Ewha-18278 = test material, OVX = ovariectomy, FL = failure load by three points bending test, ^a^p < 0.01 as compared with sham control by LSD test. ^b^p < 0.01 as compared with OVX control by LSD test. ^c^p < 0.05 as compared with sham control by MW test. ^d^p < 0.05 as compared with OVX control by MW test.

The potential of Ewha-18278 as a novel anti-osteoporotic agent in OVX-induced osteoporotic ddY mice was assessed. Ewha-18278 applied at 2.5, 5, 10 and 20 mg/kg in a volume of 10 ml/kg using 10% N, N-dimethylacetamide, along with 10% Tween 80 and 80% distilled deionized water as a vehicle was orally administered once a day for 28 days to OVX-induced osteoporotic ddY mice beginning 4 weeks after OVX. Risedronate sodium (2.5 mg/kg) served as a reference compound. Administration of Ewha-18278 into the mice was not affected body, tibia and femur weight (Supplementary Fig. S7, Supplementary Table S3, and S4). Histopathology and histomorphometrical analyses including trabecular bone volume (BV/TV), trabecular bone number (Tb.N), trabecular bone length (Tb.Le), trabecular bone thickness (Tb.Th), cortical bone thickness (Ct.Th), OC number (N.Oc), bone mineral density (BMD) and bone strength (failure load, FL) were conducted^19^,^20^. OVX mice showed decreased histomorphometrical indices including BV/TV, Tb.N, Tb.Le, Tb.Th and Ct.Th, leading to weakness of bone strength. Concentration-dependent oral administration of Ewha-18278 (5, 10 and 20 mg/kg/day) for 28 days to OVX mice led to recovery of TV/BV, Tb.N, Tb.Le, Tb.Th and Ct.Th, resulting in increased bone strength (Fig. 5A, Tables 1 and 2).

OVX mice displayed significantly reduced BMD and enhanced OC number in tibia and femur, compared to control mice. The BMD of femur in OVX control mice was significantly (p < 0.01) decreased as compared with sham control mice, but dramatic increased BMD of femur were detected in Ewha-18278 compound treated mice compared to OVX control (Fig. 5B). Moreover, treatment of Ewha-18278 resulted in decreased number of OCs in the tibia and femur, compared to control mice (Tables 1 and 2). On the other hand, the number of OBs was increased in Ewha-18278 compound-treated tibia and femur of mice compared to OVX control mice (Supplementary Fig. S8).

The bone strengths (FL) of femur and tibia mid-shaft regions in OVX control were significantly decreased compared to sham control, respectively (Fig. 5C). However, bone strength of femur and tibia were enhanced in Ewha-18278 treated mice compared to control OVX. Mice treated with risedronate did not show any favorable effects on the bone strength of femur and tibia (Fig. 5C). Overall, the anti-osteoporotic effects of Ewha-18278 (10 mg/kg/day for 28 days) were similar than those of risedronate (2.5 mg/kg/day for 28 days) in the mice. Interestingly, this concentration of Ewha-18278 was associated with increased cortical bone thickness leading to enhanced bone strength enhancement, which was did not observed for risedronate (Tables 1 and 2). Thus, these results demonstrate that Ewha-18278 targeting Nox isozyme serves as an anti-osteoportic agent through downregulation of OC number and upregulation of bone strengths.

## Discussion

Osteoporosis and its therapeutic agents have increasingly become the center of interest as the populations in advanced countries ages. The treatment of bone diseases is an approximately 11.4 billion dollar market globally, and will continue to grow; hence, the academic and commercial interest in therapeutic agents for the treatment of bone diseases^21^. In advanced countries, an estimated 4.5% of males have osteoporosis and 19.8% of females suffer from the same disease. These results suggest that osteoporosis is a more common disease than diabetes or cardiovascular diseases. Considering the suffering of patients due to fractures or when estimating costs incurred for the treatment of a disease, osteoporosis is a very important public health problem^21^. Aging is the most important risk factor for bone loss in both men and women. Aging-induced bone loss is inversely related to estrogen levels^22^ and in aging mice decreased bone formation is associated with increased ROS formation^23^. Interestingly, gonadectomy in mice and rats increases ROS level and induced bone loss and supplementation with the sexual steroids was sufficient to prevent this effect^24^. Additionally, Lean *et al.*^25^ found a crucial role of ROS in bone loss of ovarectomized mice as treatment of these animals with polyethylglycol-bound catalase inhibited bone loss.

**Figure 6:**
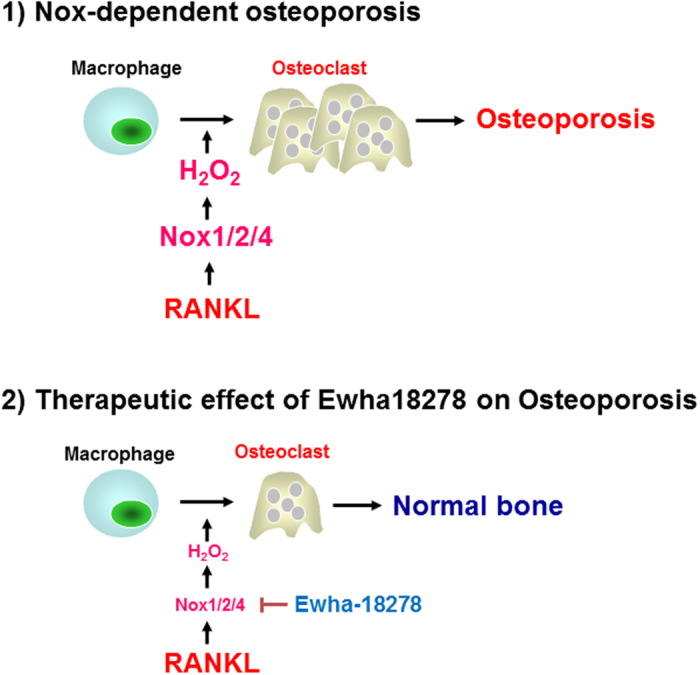
Therapeutic function of Ewha-18278 in osteoclastogenesis. RANKL stimulates Nox-dependent ROS generation leading to induction of osteoclast differentiation. Treatment of BM-derived macrophage with Ewha-18289 resulted in suppressed ROS generation and osteoclastogenesis.

Osteoporosis is known to be induced by an imbalance of bone remodeling which is mediated by a balance between OCs and OBs. Oxidative stress is involved in osteoclastogenesis. The generation of ROS is coupled with differentiation and activation of OCs^13^. Although BMMs dominantly express Nox2 isozyme, RANKL stimulates Nox1 and Nox4 expression. RANKL mediates ROS generation and OC differentiation through Nox1 activation. It indicates that that RANKL-induced osteoclastogenesis requires ROS production in osteoclast precursors via the ROS producing factor Nox1^15^. Moreover, knockdown of Nox1 expression by tranfection of Nox1-specific siRNA blocked RANKL-mediated osteoclastogenesis as well as activation of JNK or p38 mediated by RANKL. However, Nox2 knockdown had no effects on the ROS production and osteoclastogenesis induced by RANKL. More recently, Kawai *et al.* demonstrated siRNA targeting either p22^phox^ or p67^Phox^ as Nox2 regulator into RAW264.7 cells decreased ROS production and suppressed the RANKL-induced osteoclastogenesis^26^. Recently, Nox4 deficient mice show higher bone density and reduce OCs indicating that that Nox4 serves as one of the main factors for osteoclast differentiation^17^. Therefore, these results implicate Nox1, Nox2 and Nox4 as potential therapeutic targets for the treatment of osteoporosis (Fig. 6). This implies that drugs that target individual Nox enzymes will have partial effects. Thus, Nox1/2/4 inhibitor is likely to be a more effective agent for the treatment of osteoporosis.

It is well established that MAPKs and NF-kB play important roles in osteoclast differentiation^10^ and that the NFATc1 is a master transcription factor of osteoclast differentiation^27^. Moreover, activation of Nox isozymes and generation of ROS are required the osteoclastogenesis^15^,^28^. Inhibition of Nox activation and ROS generation blocked osteoclast differentiation and MAPK activation. It is widely believed that application of ROS scavengers or inhibitors of RANKL-induced ROS generating pathways represent viable alternative therapeutic strategies for bone diseases. In this report, we demonstrated that Ewha-18278 inhibits RANKL-dependent ROS generation and osteoclast differentiation (Fig. 3). Moreover, we have shown that the compound blocked the RANKL-induced activation of signaling pathways, including MAPKs and NF-kB as well as the expression of differentiation marker genes such as NFATc1 and Atp6v0d2 (Fig. 4). Our data emphasize that the inhibitory outcomes of osteoclast differentiation by the compound are mechanistically linked to the inhibition of RANKL-Nox-ROS signaling.

Nox isozymes are involved in host defense system and various cell signaling for development and homeostasis^29^,^30^. Neutrophil and macrophage expressed Nox2 isozyme preludes the ROS generation, which kills infecting microbial cells. Genetic loss of Nox2 underlies chronic granulomatous disease (CGD)^31^. Mutation of Nox3 and NoxO1 results in impaired otoconia formation in the inner ear leading to balance disorder^32^,^33^. These observations indicate that over-inhibition of Nox enzymes will have a detrimental effect. This is reminiscent of protein kinase inhibitor development for cancer therapy, in which very high affinity protein kinase inhibitors had major toxic side effects. This lesson from protein kinase inhibitor development prompted us to seek and develop pan-Nox inhibitors with moderate Ki values.

The estrogen-deficient OVX osteoporosis ddY mouse model is useful for the evaluation of osteoporotic drugs, because several parameters are clearly decreased by ovariectomy within 4–6 weeks after operation. The effects of a drug can be evaluated by examining bone weight, BMD, bone mineral content, bone strength and histomorphometrical changes of bone^19^,^20^,^34^,^35^. Using this model, the efficacy of Ewha-18278 was compared with risedronate. Risedronate is a pridinyl bisphosphonate that binds to bone hydroxyapatites and inhibits OC-mediated bone resorption^36^. Risedronate prevents bone resorption by changing OC cyctoskeleton proteins, and is effective in treatment and prevention of postmenopausal osteoporosis in women^37^,^38^. Administration of risedronate sodium (2.5 mg/kg/day for 8 weeks) in OVX rats maintains microstructural and biomechanical features of bone^39^,^40^. Therefore, presently risedronate sodium was orally adminstrated at 2.5 mg/kg/day, once daily for 28 days beginning 4 weeks after OVX. The 28-day continuous oral treatment of over 5 mg/kg of Ewha-18278 preserved bone mass and bone strength in OVX mice, indicating the potential of Ewha-18278 as a novel effective anti-osteoporosis agent (Fig. 5, Tables 1 and 2). The optimal effective dosage is considered to be 10 mg/kg, given the somewhat lower efficacy of 20 mg/kg. This may reflect absorption saturation. Very low amounts of Ewha-18278 (2.5 mg/kg) did not produce obvious favorable anti-osteoporotic effects. It is notable that Ewha-18278 significantly increased cortical bone thickness compared to risedronate (Tables 1 and 2). The increased thickness may explain the favorable bone strength enhancement effects of Ewha-18278. Interestingly, we failed to find Ewha-18278-mediated induction of OBs differentiation *in vitro* (Supplemental Fig. S6). However, the number of OBs was increased in Ewha-18278-treated mice (Supplemental Fig. S8). How Ewha-18278 affects cortical bone thickness and the OB number in mice remains to be determined. One potential hypothesis is that ovariectomy increases cellular ROS level, thereby promoting bone resorption and blocking bone formation leading to eventually induction of osteoporosis. Inhibition of Nox activity by Ewha-18278 regulates the cellular ROS level and inhibits OC differentiation in osteoporotic bone, leading to achieve a normal balance between OCs and OBs. Hence, the therapeutic activity of Ewha-18278 with increased thickness of cortical bone and the number of OBs leading to enhanced bone strength may occur in a pathological condition like ovariectomy (Fig. 6).

Our data indicate that Ewha-18278 inhibits osteoclast differentiation *in vitro* and enhanced cortical bone density and decreased osteoclast number *in vivo*. Risedronate is a bisphosphonate used to strengthen bone and prevent osteoporosis. Bisphosphonate acts act on the cholesterol biosynthesis pathway enzyme, farnesyl diphosphate synthase. By inhibiting this enzyme in the osteoclast, they interfere with geranylgeranylation (attachment of the lipid to regulatory proteins), which causes cell death in osteoclasts^41^,^42^. This mechanism is responsible for the bisphosphonate-mediated suppression of osteoclastic bone resorption and reduction of bone turnover contributing to fracture prevention. Since these two compounds have different modes of action, these two compounds may show synergistic effects.

Many therapeutic agents have been discovered and marketed. Bisphosphonate (Fosamax™) and its derivatives are well-known agents for treatment of osteoporosis^43^. However, these compounds have serious side effect on due to their very low bioavailability^44^. Soluble estrogen receptor modulator (Raloxifene™) for estrogen replacement increases bone density by 3–4%, but carries the potential risk of breast cancer^45^. Parathyroid hormone therapy is a novel approach for the activation of OBs to balance bone remodeling. However, this therapy has a risk of osteosarcoma^46^. Recently, the humanized monoclonal antibody to RANKL (Denosumab; Prolia™) has become available. The expected side effect of this therapy is immune deficiency due to RANKL scavenging^47^. Ewha-18278 blocks Nox isozymes as a part of the RANKL signaling cascade of OC differentiation. Therefore, Ewha-18278 treatment should have not overly disrupted RANKL signaling in immune responses. Moreover, Ewha-18278 has good characteristics, such as very high bioavailability and specific mode of action, for development of therapeutic agent in osteoporosis.

In conclusion, we identified a novel pyrazol derivative, Ewha-18278, through a structure-activity analysis of compounds screened from a chemical library. Ewha-18278 inhibits Nox isozymes and attenuates RANKL-mediated OC differentiation. Oral administration of Ewha-18278 to OVX mice increases histomorphometrical indices including BMD, BV/TV, Tb.N, Tb.Le, Tb.Th and Ct.Th, leading to enhanced bone strength. Ewha-18278 is a therapeutic drug candidate for treatment of osteoporosis (Fig. 6).

## Methods

### Synthesis of 3-phenyl-1-(pyridin-2-yl)-1*H*-5-hydroxypyrazole (Ewha-89403)

A flame-dried 100 mL round bottom flask was charged with 2-hydrazinopyridine (10.9 g, 100 mmol) and ethyl benzoylacetate (19.2 g, 100 mmol), and was heated to 160 °C for 12 hours in neat condition under argon atmosphere. The crude product was purified by silica gel column chromatography (hexane/EtOAc = 7/3) to afford title compound as a yellow solid, which was recrystallized in MeOH to give light yellow crystals (20.6 g, 87% yield); mp: 122.6–123.2 ^o^C; ^1^H NMR (500 MHz, CDCl_3_) δ 12.87 (brs, 1H), 8.25–8.23 (ddd, *J* = 0.7, 1.6, 5.0 Hz, 1H ), 8.04 (d, *J* = 8.3 Hz, 1H), 7.93–7.91 (td, *J* = 1.4, 8.4 Hz, 2H), 7.87–7.84 (ddd, *J* = 1.8, 7.5, 9.2 Hz, 1H), 7.48–7.46 (dt, *J* = 1.3, 6.4 Hz, 2H), 7.42–7.38 (m, 1H), 7.13–7.10 (ddd, *J* = 1.0, 5.1, 7.3 Hz, 1H), 6.00 (s, 1H); ^13^C NMR (125 MHz, CDCl_3_) δ 157.3, 154.5, 152.6, 145.1, 139.9, 133.1, 128.6, 128.5, 125.9, 120.0, 112.2, 85.7; HRMS (EI) *m/z* calc’d for C_14_H_11_N_3_O, 237.0902; found 237.0898 (Supplemental Fig. 1). Atomic coordinates and crystallographic parameters of Ewha-89403 have been deposited at the Cambridge Crystallographic Data Centre (CCDC 805262). These data can be obtained free of charge from the Cambridge Crystallographic Data Centre via www.ccdc.cam.ac.uk/data_request/cif.

### Synthesis of 3-phenyl-1-(pyridin-2-yl)-4-propyl-1*H*-5-hydroxypyrazole HCl (Ewha-18278)

A flame-dried 100 mL round bottom flask was charged with 2-hydrazinopyridine (10.9 g, 100 mmol) and ethyl 2-benzoylpentanoate (23.4 g, 100 mmol), and was heated to 160 ^o^C for 12 hours in neat condition under argon atmosphere. The crude product was purified by silica gel column chromatography (hexane/EtOAc = 8/2) to afford title compound as a white solid, which was recrystallized with hexane/Et_2_O to give colorless needles (23.5 g). To a solution of this solid in anhydrous Et_2_O (200 mL) was added HCl (107 mL, 1 *M* in Et_2_O) using syringe for 30 minutes at 0 ^o^C. The resulting mixtures were stirred for 2 hours at the same temperature. The precipitate was filtered off and then washed with ice-cold ether (300 mL) to give title compound as a white powder (24.0 g, 76%); mp: 156.8–157.5 ^o^C; ^1^H NMR (300 MHz, DMSO-*d*_6_) δ 11.54 (brs, 2H), 8.46 (d, *J* = 4.6 Hz, 1H ), 8.09 (brs, 2H), 7.67 (d, *J* = 7.3 Hz, 2H), 7.51–7.35 (m, 4H), 2.48 (t, *J* = 7.0 Hz, 2H), 1.49 (septet, *J* = 7.0 Hz, 2H), 0.85 (t, *J* = 7.3 Hz, 3H); ^13^C NMR (125 MHz, DMSO-*d*_6_) δ 156.2, 150.9, 150.5, 146.0, 140.8, 132.1, 128.7, 128.6, 127.5, 120.7, 112.4. 101.9, 23.8, 22.2, 13.7. (Supplemental Fig. 2).

### Nox inhibitor screening

Nox1, 2, or 4 inhibitory activities were determined in a novel assay involving synthetic compounds along with the hydroxypyrazole derivative as a reference. To specifically inhibit Nox isozymes, transgenic *Drosophila* Duox knockdown lines were established that over-expressed human Nox1, 2, or 4^48^. The genotypes used were human Nox1 (*UAS-hNOX1/UAS-DUOX-RNAi; Da-GAL4*/+), Nox2 (*UAS-hNOX2/UAS-DUOX-RNAi; Da-GAL4*/+) and Nox4 (*UAS-hNOX4/UAS-DUOX-RNAi; Da-GAL4*/+). Transgenic flies were homogenated with ice-cold PBS containing protease inhibitors and membrane-enriched human Nox1, 2, or 4 were harvested. Membranes containing human Nox1, 2, or 4 served to monitor ROS production by lucigenin chemiluminescence in the absence or presence of chemical compound^18^. The reaction medium consisted in Hepes-buffered salt solution (145 mM NaCl, 4.8 mM KCl, 1.2 mM MgSO_4_, 1.0 mM KH_2_PO_4_, 1.75 μM CaCl_2_, 0.03 Na2EDTA, 5.5 mM glucose, and 10 mM HEPES, pH 7.4), 400 μM lucigenin (10,10-dimethyl-bis-9,9-bisacridinium nitrate) and 500 μM NADPH.

### Cell culture and OC differentiation

Primary BMMs were obtained from 6- to 8-week-old male C57BL/6 mice (The Jackson Laboratory, Bar Harbor, MN, USA) as described previously^49^. In brief, bone marrow cells were cultured in α-MEM (HyClone, Logan, UT, USA) containing 10% fetal bovine serum (FBS) (HyClone, Logan UT, USA) with 30 ng/ml macrophage-colony stimulating facor (M-CSF) for 3 days to obtain OC precursor cells of monocyte/macrophage lineage. For OC differentiation, the precursor cells were treated with 100 ng/ml RANKL in the presence of M-CSF with or without for 3–4 days. OCs were fixed with 4% paraformaldehyde and stained for tartrate-resistant acid phosphatase (TRAP) using a leukocyte acid phosphatase cytochemistry kit (Sigma-Aldrich, St. Louis, MO, USA) according to the manufacturer’s instructions. TRAP-positive multinucleated cells (TRAP^+^ MNCs) containing three or more nuclei were counted as OC-like cells.

### Bone resorption assay

Mature OCs incubated with 100 ng/ml RANKL for 3 days were seeded onto dentine discs (Immunodiagnostic Systems, Gaithersburg, MD, USA) and further incubated in the presence of 100 ng/ml RANKL with Ewha-18278 (10 μM) or DMSO for 24 hours. The cells were removed from the dentine discs and the dentine discs were stained with hematoxylin. Pit areas were photographed under a light microscope and analyzed using Image-Pro Plus version 4.5 software (Media-Cybernetics, Rockville, MD, USA).

### OC fusion assay

Pre-OCs were generated with M-CSF and RANKL, then treated with Ewha-18278 (10 μM) or DMSO for 24 hours in the presence of M-CSF and RANKL. Cells were stained for TRAP using a leukocyte acid phosphatase cytochemistry kit (Sigma-Aldrich, St. Louis, MO, USA) according to the manufacturer’s instructions.

### TdT-UDP nick end labeling (TUNEL) assay

Apoptotic cells were detected by the TdT-UDP nick end labeling (TUNEL) assay *In Situ* Cell Death Detection Kit (Roche Applied Science, Basel, Switzerland) according to the manufacturer’s instructions. In brief, mature OCs were placed in fresh medium containing 100 ng/ml RANKL and cultured for 6 hours with Ewha-18278 or DMSO. The percentage of positively stained cells was determined by counting the numbers of TUNEL labeled and DAPI stained cells. The experiments were repeated 3 times, and the mean values were calculated.

### Real-time PCR analysis

BMMs were cultured with or without Ewha-18278 for the indicated periods. Total RNA was extracted using TRIzol reagent (Invitrogen, Carlsbad, CA, USA) according to the manufacturer’s instructions. After denaturation of total RNA at 70 °C for 10 minutes, first-strand cDNA was synthesized with oligo (dT) primers and MMLV-reverse transcriptase (SolGent, Seoul, Korea). The relative mRNA levels were evaluated by real-time PCR using a SYBR Green Master kit (Kapa Biosystems, Woburn, MA, USA) and reactions were performed in triplicate on ABI PRISM 7300 unit (Applied Biosystems, Franklin Lakes, NJ, USA). PCR primers were: *NFATc1* sense, 5′-CCAGAAAATAACATGCGAGCC-3′; antisense, 5′-GTGGGATGTGAACTCGGAAG-3′; *Atp6v0d2* sense, 5′-CAGAGATGGAAGCTGTCAACATTG-3′; antisense, 5′-TGCCAAATGAGTTCAGAGTG-3′; *β-actin* sense, 5′-ACCCTAAGGCCAACCGTG-3′; antisense, 5′-GCCTGGATGGCTACGTAC-3′. Melting curve analysis and agarose gel electrophoresis were performed to ensure a single PCR product. The relative expression levels were normalized to the level of actin mRNA as an internal control gene.

### Western blot analysis

BMMs were cultured with or without Ewha-18278 for the indicated periods. Equal amounts of proteins in cell lysates were separated by SDS-PAGE and electrotransferred to a PVDF membrane (Millipore, Billerica, MA, USA). The membrane was blocked in PBS containing 5% nonfat dry milk for 1 hour and then immunoblotted overnight at 4 °C on a shaker with antibodies to phospho-ERK, phospho-p38, phospho-JNK, phospho-IκBα, ERK, p38, JNK, IκBα (all from Cell Signaling Technology, Danvers, MA), NFATc1, and β-actin (both from Santa Cruz, CA, USA) in Tris-buffered saline containing 0.05% Tween 20 (TBS-T) with 1% bovine serum albumin (BSA). Following washes in TBS-T, the membrane was incubated with either horseradish peroxidase-conjugated anti-rabbit antibody or anti-mouse antibody (Thermo Scientific, Pittsburg, PA, USA) in TBS-T for 1 hour at room temperature. Proteins were detected using an ECL detection kit (Amersham Biosciences, Piscataway, NJ, USA). Anti-Atp6v0d2 antibody was kindly provided by Y. Choi (University of Pennsylvania).

### Detection of intracellular ROS

BMMs were pretreated with or without Ewha-18278 for 1 hour, stimulated with RANKL for 10 minutes, and the cells were washed with α-MEM lacking phenol red and then incubated with 10 μM of 2’, 7’-dichlorofluorescin diacetate (DCF-DA) in the dark at 37 ^o^C (for 10 minutes). DCF-DA is oxidized by ROS to the highly fluorescent DCF. After washed with HBSS, the cells were then examined with a laser scanning confocal microscope (LSM510; Zeiss) equipped with an argon laser tuned to an excitation wavelength of 488 nm, an LP505 emission filter (515–540 nm), and a Zeiss Axiovert objective lens. Images were digitized and stored at a resolution of 512 by 512 pixels. Five groups of cells were randomly selected from each sample, and the mean relative fluorescence intensity for each group of cells was measured with a Zeiss vision system and then averaged for all groups. All experiments were repeated at least three times.

### Histomorphometrical analysis

The study was reviewed and approved by the Institutional Animal Care and Use Committee (IACUC) of Center for Laboratory Animal Sciences, Ewha Industry-University Cooperation Foundation, Ewha Womans University. Experimental methods were carried out in accordance with the approved ethic guidelines in Ewha Industry-University Cooperation Foundation, Ewha Womans University. Beginning 4 weeks after OVX, the female ddY mice received 2.5, 5, 10, or 20 mg/kg of #18–278, 2.5 mg/kg of risedronate, or vehicle once daily for 28 days. The Changes in body weight, bone weight, bone mineral density (BMD), failure load (FL), bone calcium (Ca), inorganic phosphorus (P) contents, histology and histomorphology were monitored. Serum osteocalcin and bone specific alkaline phosphatase (bALP) were monitored in mice receiving Ewha-18278.

### Estimation of osteoporotic indexes in OVX

BMD (g/cm^2^) of total right femur was detected using dual-energy x-ray absorptionmetry (DEXA) using a PXImus apparatus (Lunar, Madison, WI, USA). Bone strength was detected as failure load. The failure load of right tibia mid-shaft was detected (Newton, *N*) by a three-point bending test using a model 6022 computerized testing machine (Instron, Canton, OH, USA) operating at a speed of 20 mm/min.

### Estimation of ash bone and bone Ca and P contents

The right sides of femur and tibia were weighed and then dried at 120 °C for 8 hours. The dried bones were carbonized at 800 °C for 6 hours in a furnace (Daihan Scientific, Korea) to detect ash absolute weights, after measurement of BMD and failure load. To reduce the individual body weight differences, the relative weight (%) was calculated using body weight at sacrifice and absolute weight as [Relative bone weights = (Absolute bone weight/Body weight at sacrifice) ×100]. In addition, ashed bones were ground and dissolved in nitric acid. In diluted solution, Ca and P contents were calculated (mg/g bone) using an orthocresolphthalein complex and enzyme methods, respectively. The Ca/P ratio (%) was calculated as (Tibia Ca contents/Tibia P contents) ×100.

### Histological analysis

All mice were euthanized 28 days after the first treatment of Ewha-18278. The left side of femur of each mouse was separated and fixed in 10% neutral buffered formalin and decalcified in a solution comprised of 24.4% formic acid and 0.5N sodium hydroxide for 5 days, with spent solution replaced with fresh solution each day. Each sample was embedded in paraffin, sectioned to a thickness of 3–4 μm and exposed to with Hematoxylin & Eosin (H&E) stain. Histomorphometry was conducted as for bone mass and structure with bone resorption in a uniform area of epiphyseal regions of femur (growth plate regions were excluded). Cortical bone thickness was measured in the mid-shaft region of the femur.

### Docking simulation of Nox inhibitors with gp91^phox^

To simulate how Ewha-89403 and Ewha-18278 inhibits the NADPH oxidase isozymes, crystal structure of NADPH binding domain of gp91^phox^/Nox2 (PDB ID: 3A1F) was utilized. NADPH binding domain of gp91^phox^/Nox2 is highly conserved in Nox1 and Nox4. To find the most likely binding pocket of the inhibitors, the NADPH binding domain was placed on the grid points and mapped according to its binding site properties including hydrophobicity and hydrophilicity. The best site was selected based on SiteScore which was the sum of the site size score, enclosure score and hydrophilic score.

### Phagocytosis assay

BMMs were pretreated with Ewha-18278 NOX inhibitor for 1 hour, and the cells were incubated with FITC-zymosan (Molecular Probes) for 30 min at 37 °C. After the incubations, cells were washed three times with PBS, and the quantities of internalized fluorescent particles were determined by flow cytometry. FITC-zymosan uptake was quantified as mean fluorescence intensity (MFI).

### Glucose oxidase assay

Glucose oxidase activity was measured by lucigenin. Reaction mixtures contained 0.75 mM glucose, 0.25 mg/ml glucose oxidase, and 0.4 mM lucigenin with the indicated inhibitor (0–20μM). Luminescence was measured over 30 min at 37 °C with luminometor (DTX800, Beckman coulter).

### Xanthine oxidase assay

Xanthine oxidase activity was measured by lucigenin. Reaction mixtures contained 0.1 mU xanthine oxidase, 0.25 mM Xanthine, and 0.4 mM lucigenin with the indicated inhibitor (0–20 μM). Luminescence measured over 30 min at 37 °C with luminometor (DTX800, Beckman coulter).

### Statistics

Data are expressed as the means ± standard deviation (SD) or ± standard error of the values from three to five independent experiments. Statistical analysis was performed with a two-tailed Student’s t-test. P value of <0.05 was considered statistically significant.

## Additional Information

**How to cite this article**: Joo, J. H. *et al.* A novel pyrazole derivative protects from ovariectomy-induced osteoporosis through the inhibition of NADPH oxidase. *Sci. Rep.* **6**, 22389; doi: 10.1038/srep22389 (2016).

## Supplementary Material

Supplementary Information

## Figures and Tables

**Table 1:** Tibia histopathology-histomorphometry in OVX Mice.

Groups	Bone mass and structure					Bone resorption
	BV/TV (%)	Tb.N (numbers)	Tb.Le (mm)	Tb.Th (μm)	Ct.Th (μm)	N.OC/BS(numbers)
Controls						
Sham	37.74 ± 5.38	17.50 ± 1.60	1.09 ± 0.13	68.14 ± 10.06	195.35 ± 16.98	3.00 ± 1.20
OVX	18.17 ± 1.61^a^	7.88 ± 1.25^a^	0.39 ± 0.07^a^	33.05 ± 4.31^a^	117.35 ± 11.22^a^	15.13 ± 3.09^a^
Risedronate	35.71 ± 5.63^c^	19.25 ± 1.67^bc^	0.76 ± 0.12^ac^	43.52 ± 8.73^ac^	113.81 ± 14.45^a^	19.38 ± 3.20^ac^
Ewha-18278						
20.0mg/kg	33.78 ± 3.47^c^	17.13 ± 1.81^c^	0.83 ± 0.17^ac^	55.98 ± 7.15^ac^	194.28 ± 15.50^c^	6.88 ± 2.36^ac^
10.0mg/kg	33.36 ± 4.35^c^	15.00 ± 2.00^ac^	0.78 ± 0.15^ac^	53.36 ± 5.94^ac^	182.11 ± 12.48^c^	8.13 ± 2.23^ac^
5.0mg/kg	29.39 ± 2.41^bc^	12.50 ± 1.31^ac^	0.72 ± 0.12^ac^	48.34 ± 6.87^ac^	137.51 ± 10.96^ac^	
2.5mg/kg	19.04 ± 3.10^a^	8.63 ± 1.60^a^	0.41 ± 0.08^a^	34.89 ± 5.16^a^	122.04 ± 11.96^a^	14.13 ± 3.44^a^

Values are expressed mean ± S.D. of eight mice.

Risedronate sodium was administered at 2.5mg/kg levels.

^a^p < 0.01 and ^b^p < 0.05 as compared with sham control by LSD test.

^c^p < 0.01 as compared with OVX control by LSD test.

Ewha-18278 = test material.

OVX = ovariectomy.

BV/TV = Trabecular bone volume (%).

Tb.N = Trabecular bone number (N/epiphyseal).

Tb.Le = Trabecular bone length (Longitudinal thickness; mm).

Tb.Th = Trabecular bone thickness (Cross thickness; μm).

Ct.Th = Cortical bone thickness (Cross thickness; μm).

N.OC/BS = Osteoclast cell number (N/epiphyseal).

**Table 2:** Femur histopathology-histomorphometry in OVX Mice.

Groups	Bone mass and structure					Bone resorption
	BV/TV (%)	Tb.N (numbers)	Tb.Le (mm)	Tb.Th (μm)	Ct.Th (μm)	N.OC/BS(numbers)
Controls						
Sham	37.80 ± 5.02	20.75 ± 2.66	1.19 ± 0.10	88.79 ± 10.65	187.82 ± 17.85	4.00 ± 1.20
OVX	21.26 ± 2.55^a^	9.63 ± 1.41^e^	0.63 ± 0.09^a^	37.18 ± 10.28^a^	130.79 ± 15.82^a^	14.88 ± 1.89^e^
Risedronate	38.08 ± 7.20^c^	24.50 ± 4.00^eg^	1.04 ± 0.12^ac^	47.25 ± 4.73^ad^	128.36 ± 15.57^a^	19.00 ± 2.93^eg^
Ewha-18278						
20.0mg/kg	34.07 ± 3.89^c^	16.63 ± 1.19^eg^	1.07 ± 0.15^bc^	71.98 ± 12.20^ac^	198.06 ± 12.51^c^	5.63 ± 1.41^fg^
10.0mg/kg	33.63 ± 3.51^c^	15.38 ± 1.60^eg^	0.95 ± 0.10^ac^	68.14 ± 11.23^ac^	165.52 ± 12.06^ac^	6.63 ± 1.30^eg^
5.0mg/kg	29.06 ± 2.72^ac^	11.25 ± 1.04^eh^	0.87 ± 0.05^ac^	52.98 ± 8.96^ac^	159.04 ± 13.45^ac^	10.63 ± 2.77^eg^
2.5mg/kg	23.62 ± 3.63^a^	9.75 ± 1.67^e^	0.68 ± 0.12^a^	40.98 ± 5.94^a^	136.48 ± 10.32^a^	13.75 ± 2.25^e^

Values are expressed mean ± S.D. of eight mice.

Risedronate sodium was administered at 2.5mg/kg levels.

^a^p < 0.01 and ^b^p < 0.05 as compared with sham control by LSD test.

^c^p < 0.01 and ^d^p < 0.05 as compared with OVX control by LSD test.

^e^p < 0.01 and ^f^p < 0.05 as compared with sham control by MW test.

^g^p < 0.01 and ^h^p < 0.05 as compared with OVX control by MW test.

Ewha-18278 = test material.

OVX = ovariectomy.

BV/TV = Trabecular bone volume (%).

Tb.N = Trabecular bone number (N/epiphyseal).

Tb.Le = Trabecular bone length (Longitudinal thickness; mm).

Tb.Th = Trabecular bone thickness (Cross thickness; μm).

Ct.Th = Cortical bone thickness (Cross thickness; μm).

N.OC/BS = Osteoclast cell number (N/epiphyseal).
