# Supplementary material for: A novel pyrazole derivative protects from ovariectomy-induced osteoporosis through the inhibition of NADPH oxidase
Source: Sci Rep. 2016 Mar 15;6:22389. doi: 10.1038/srep22389 (PMC4792161; doi:10.1038/srep22389)
Supplement: Supplementary Information [file srep22389-s1.pdf]

Supplementary Information

A novel pyrazole derivative protects from ovariectomy-induced osteoporosis through the inhibition of NADPH oxidase

Jung Hee Joo[1*], Jeong-Eun Huh[1*], Jee Hyun Lee[1], Doo Ri Park[1], Yoonji Lee[2], Seul Gee Lee[2], Sun Choi[2], Hwa Jeong Lee[2], Seong-Won Song[3], Yongmi Jeong[3], Ja-Il Goo[4], Yongseok Choi[4], Hye Kyung Baek[5], Sun Shin Yi[5], Soo Jin Park[6], Ji Eun Lee[6], Sae Kwang Ku[6], Won Jae Lee[7], Kee-In Lee[8], Soo Young Lee[1], Yun Soo Bae[1]

[1]Department of Life Science, Ewha Womans University, Seoul, Republic of Korea, [2]College of Pharmacy and Graduate School of Pharmaceutical Sciences, Ewha Womans University, Seoul 120-750, Korea, [3]Bioresearch Institute, Yooyoung Pharmaceuticals Co. Ltd., Seoul 152-719, Republic of Korea, [4]College of Life Sciences and Biotechnology, Korea University, Seoul 136-701, Korea, [5]Department of Biomedical Laboratory Science, College of Medical Sciences, Asan 31538, Soonchunhyang University, [6]Department of Anatomy and Histology, College of Oriental Medicine, and The Medical Research center for Globalization of Herbal Formulation, Daegu Haany University, Gyeongsan 712-715, Republic of Korea, [7]School of Biological Science, Seoul National University and National Creative Research Initiative Center for Symbiosystem, Seoul National University, Seoul 151-742, South Korea, [8]Green Chemistry Division, Korea Research Institute of Chemical Technology, P.O. Box 107, Yuseong, Taejon 305-600, Korea

Correspondence: Yun Soo Bae, Department of Life Sciences, Ewha Womans University, 52 Ewhayeodae-Gil, Seodaemoon-Gu, Seoul 120-750, Korea, Phone: 82-2-3277-2729, Fax: 82-2-3277-3760, e-mail: baeys@ewha.ac.kr; Soo Young Lee, Department of Life Sciences, Ewha Womans University, 52 Ewhayeodae-Gil, Seodaemoon-Gu, Seoul 120-750, Korea, Phone: 82-2-3277-3770, Fax: 82-2-3277-3760, e-mail: leesy@ewha.ac.kr; Kee-In Lee, Green Chemistry Division, Korea Research Institute of Chemical Technology, P.O. Box 107, Yuseong, Taejon 305-600, Korea, e-mail: kilee@krict.re.kr

Supplementary Figure S1

A

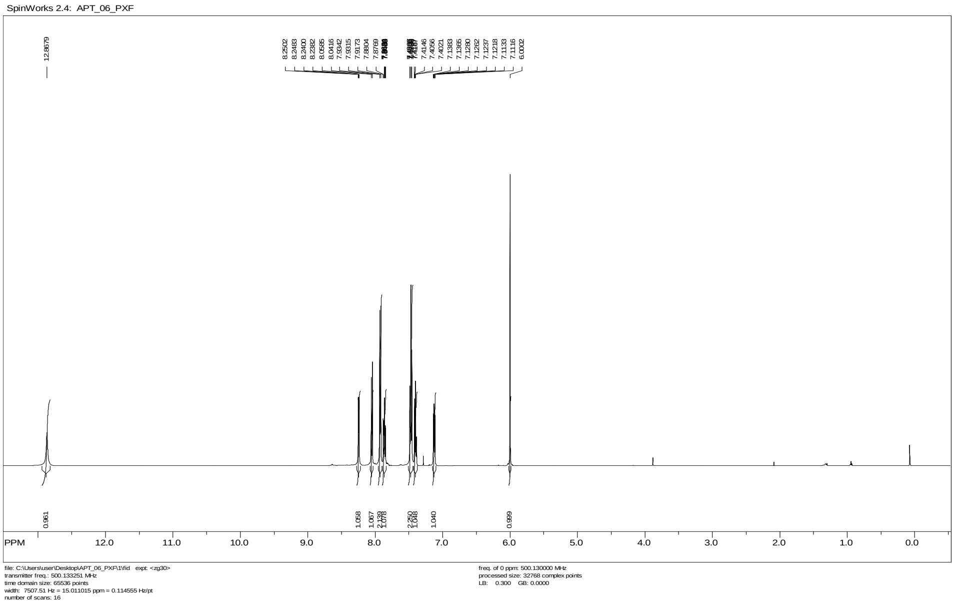

B

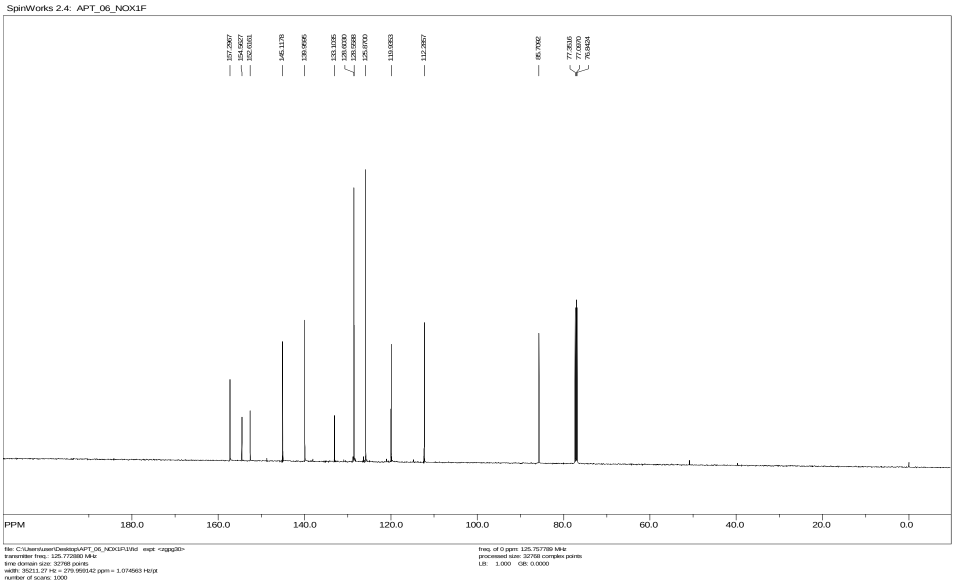

Supplementary Figure S1. The product (Ewha-89403) was purified by silica gel column chromatography (hexane/EtOAc = 7/3) to afford title compound as a yellow solid, which was recrystallized in MeOH to give light yellow crystals (Ewha-89403, 20.6 g, 87% yield); mp: 122.6-123.2 ^{o}C. **A**,^{1}H NMR (500 MHz, $CDCl_3$) δ 12.87 (brs, 1H), 8.25-8.23 (ddd, J = 0.7, 1.6, 5.0 Hz, 1H), 8.04 (d, J = 8.3 Hz, 1H), 7.93-7.91 (td, J = 1.4, 8.4 Hz, 2H), 7.87-7.84 (ddd, J = 1.8, 7.5, 9.2 Hz, 1H), 7.48-7.46 (dt, J = 1.3, 6.4 Hz, 2H), 7.42-7.38 (m, 1H), 7.13-7.10 (ddd, J = 1.0, 5.1, 7.3 Hz, 1H), 6.00 (s, 1H); **B**, ^{13}C NMR (125 MHz, $CDCl_3$) δ 157.3, 154.5, 152.6, 145.1, 139.9, 133.1, 128.6, 128.5, 125.9, 120.0, 112.2, 85.7; HRMS (EI) *m/z* calc'd for $C_{14}H_{11}N_3O$, 237.0902; found 237.0898

Supplementary Figure S2

A

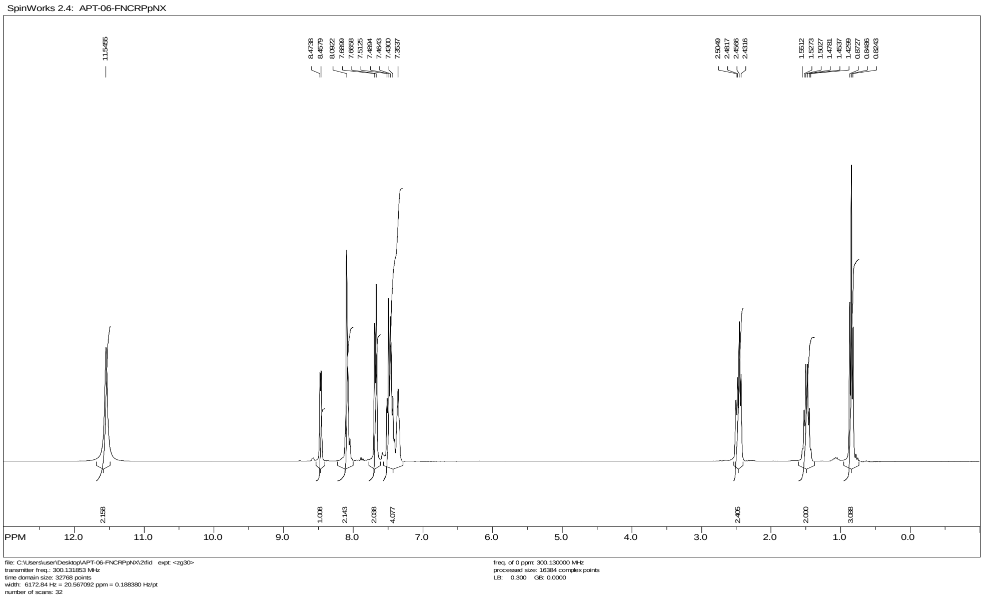

B

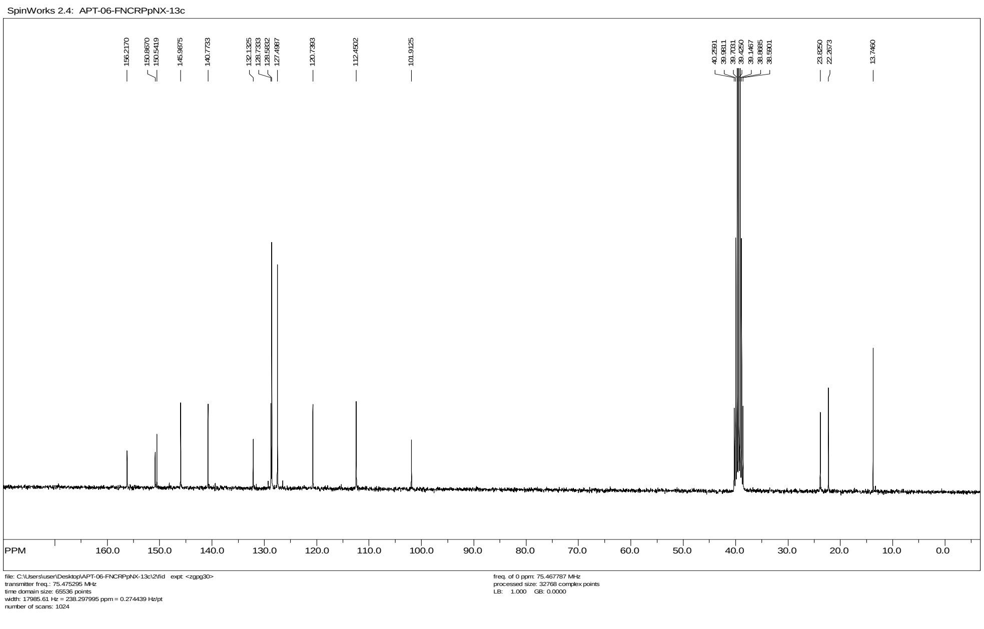

Supplementary Figure S2. The precipitate (Ewha-18278) was filtered off and then washed with ice-cold ether (300 mL) to give title compound as a white powder (24.0 g, 76%); mp: 156.8- 157.5 ^{o}C. **A**, ^{1}H NMR (300 MHz, DMSO-d_6) δ 11.54 (brs, 2H), 8.46 (d, J = 4.6 Hz, 1H), 8.09 (brs, 2H), 7.67 (d, J = 7.3 Hz, 2H), 7.51-7.35 (m, 4H), 2.48 (t, J = 7.0 Hz, 2H), 1.49 (septet, J = 7.0 Hz, 2H), 0.85 (t, J = 7.3 Hz, 3H); **B**, ^{13}C NMR (125 MHz, DMSO-d_6) δ 156.2, 150.9, 150.5, 146.0, 140.8, 132.1, 128.7, 128.6, 127.5, 120.7, 112.4. 101.9, 23.8, 22.2, 13.7.

Supplementary Figure S3

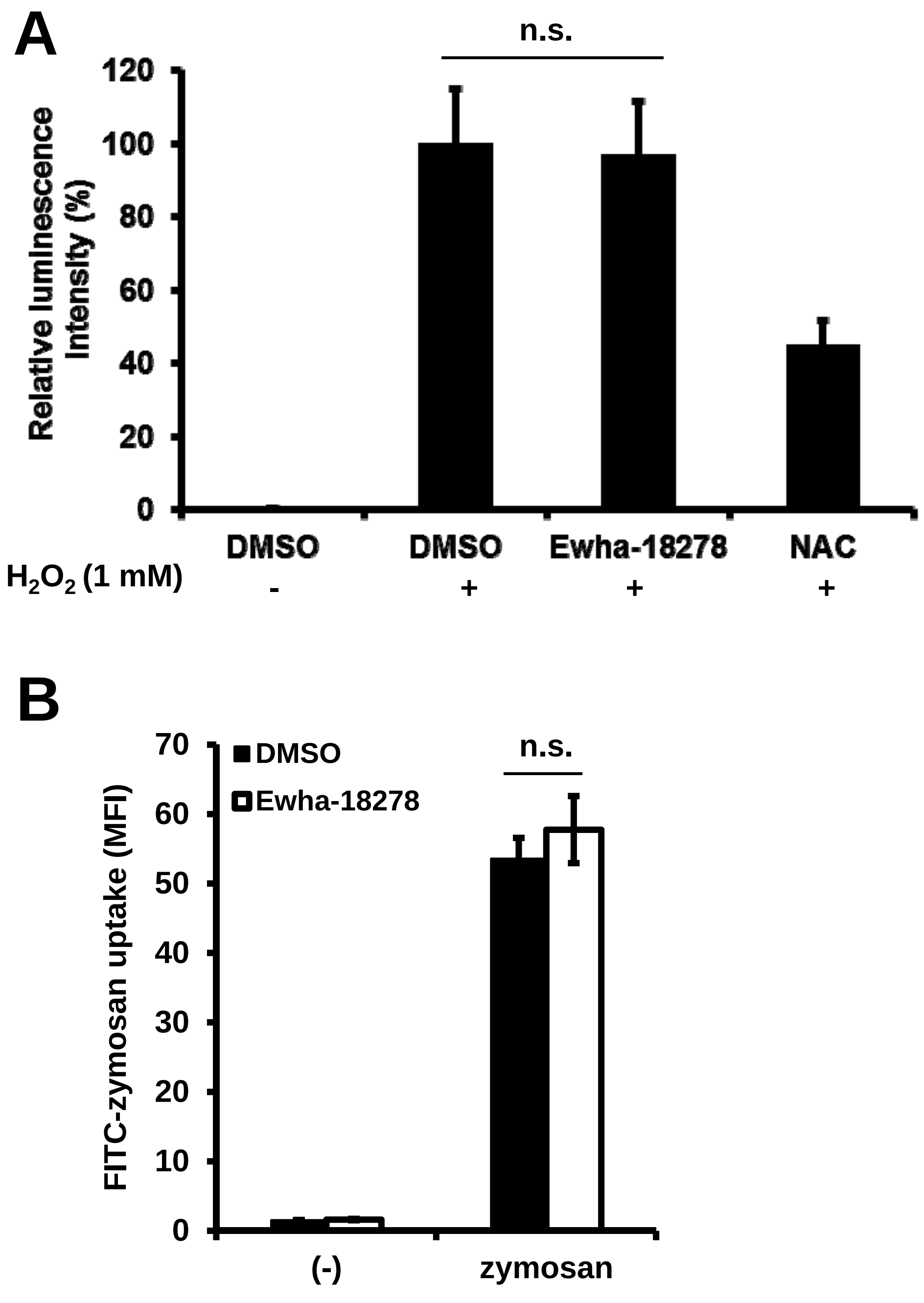

Supplementary Figure S3. (A) Ewha-18278 compounds can not directly scavenge superoxide or other reactive species in vitro. ROS (H_2O_2) measured with lucigenin. Reaction mixtures contained 1 mM H_2O_2 with Ewha-18278 (20 μM) or NAC (1 mM). Luminescence measured over 30 min at 37 °C with luminometor (Gloma X luminometor, Turner BioSystem). (B) Effect of the Ewha-18278 on the phagocytosis of zymosan. BMMs were pretreated with Ewha-18278 for 1 hour, the cells were incubated with FITC-zymosan particles, and internalized fluorescence was measured by flow cytometry. The data are presented as the MFI of internalized FITC. Data represent mean ± S.D. n.s., not significant.

Supplementary Figure S4

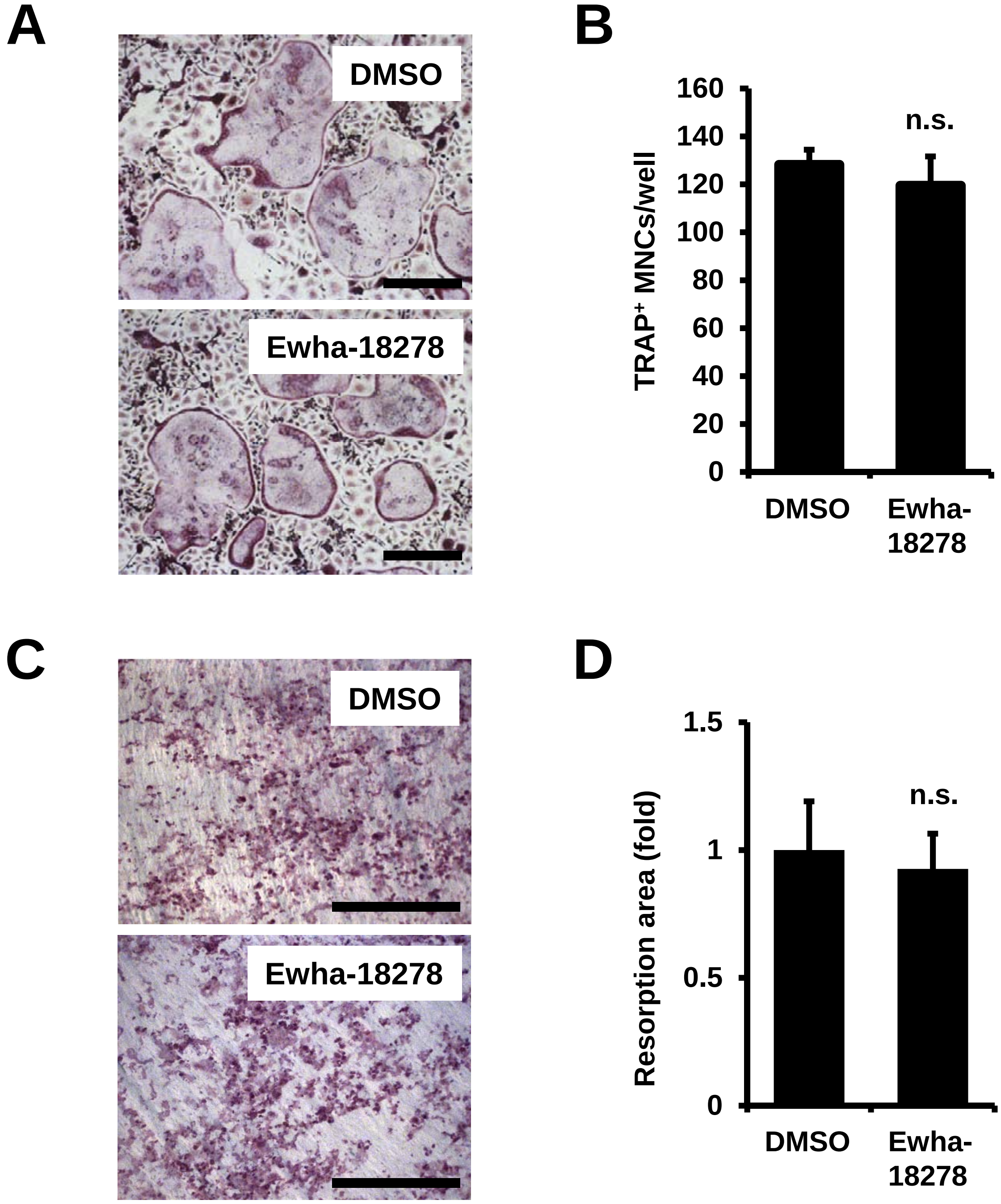

Supplementary Figure S4. Effect of Ewha-18278 on osteoclast fusion and bone resorption. (A,B) BMMs were cultured with M-CSF and RANKL for 3 days, then Ewha-18278 (10 μM) or DMSO were added in the maturation phase for 24 hours. Cells were stained for TRAP activity (A). $TRAP^+$ MNCs containing more than 3 nuclei were counted (B). Scale bar, 500 μm. (C,D) Mature OCs were generated with M-CSF and RANKL, cells were seeded onto dentine discs and further cultured with Ewha-18278 (10 μM) or DMSO for 24 hours. Cells were stained with hematoxylin for visualization of pit formation (C). The area of resorption pits were measured with Image-Pro Plus 4.5 (D). Scale bar, 500 μm. Data represent mean ± S.D., n.s., not significant.

Supplementary Figure S5

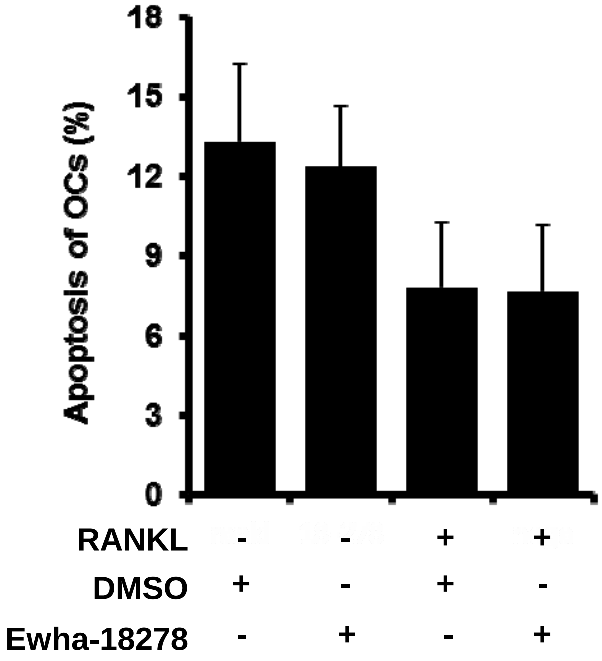

Supplementary Figure S5. Effect of Ewha-18278 on apoptosis of osteoclast. Mature OCs were generated with M-CSF and RANKL. Cells were washed with PBS and then treated with Ewha-18278 (10 μM) or DMSO with or without RANKL for 20 hours. Cells were subjected into TUNEL assay. Data represent mean ± S.D.

Supplementary Figure S6

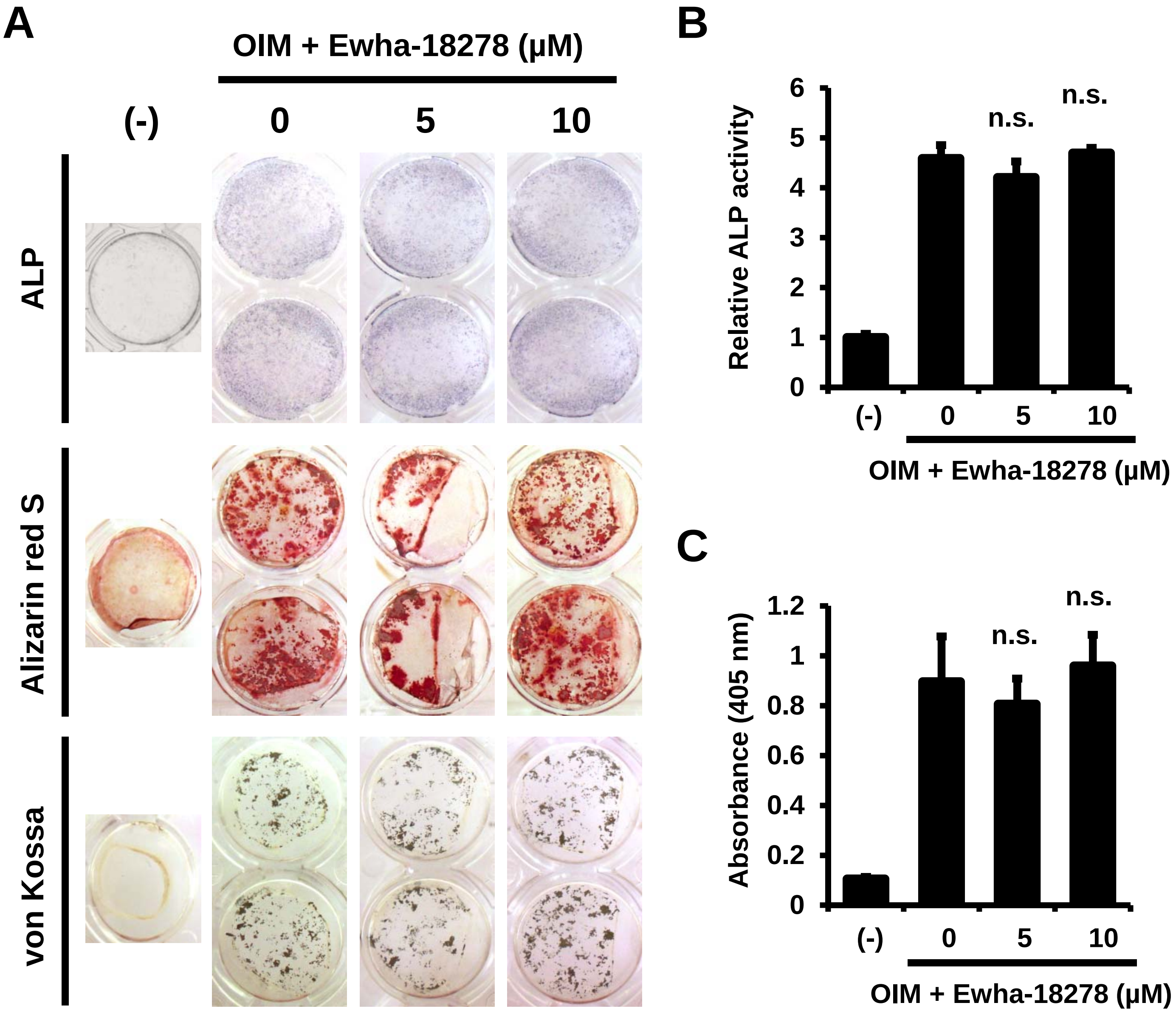

Supplementary Figure S6. The Ewha-18278 has no effects on osteoblast differentiation. Primary calvarial pre-osteoblasts were cultured with osteogenesis induction medium (OIM) with or without the indicated concentrations of Ewha-18278. ALP activity was determined by staining with NBT/BCIP staining (A, upper) and quantitative ALP enzyme activity was determined using p-nitrophenyl phosphate (pNPP) as the substrate (B). Cellular mineralization was assessed by alizarin red S (A, middle) and von Kossa (A, lower) staining, respectively. The alizarin red S was quantified at 405 nm (C). Results are representative of at least three independent experiments. n.s., not significant.

Supplementary Figure S7

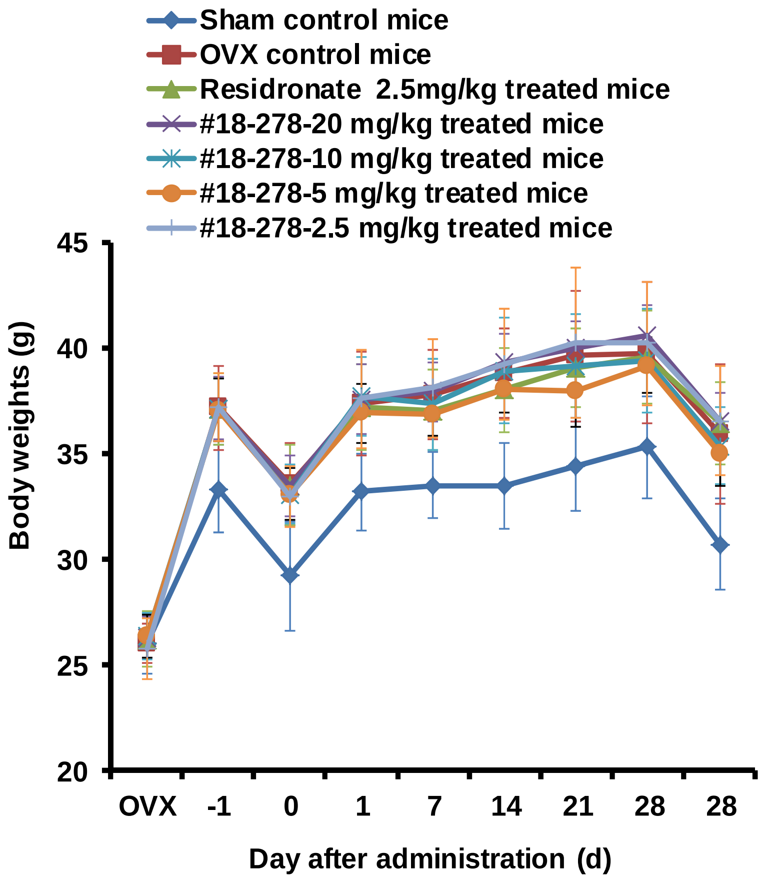

Supplementary Figure S7. Body weight changes in OVX mice. We selected eight mice per group showing more increases of body weights as compared with sham-operated mice and regarded as good OVX animals at four weeks after OVX surgery, consequently, significant increases of body weights were detected in all OVX groups as compared with sham control mice throughout 28 days of administration periods. Anyway, no meaningful changes on the body weights were detected in risedronate and Ewha-18278 2.5, 5, 10 and 20 mg/kg treated mice as compared with OVX control mice, in the present study. Values are expressed mean ± S.D. of eight mice. Risedronate sodium was administered at 2.5mg/kg levels. Ewha-18278 = test material, OVX = ovariectomy. Number -1 means 1 day before start of administration at 27 days after OVX surgery, Number 0 means at start of administration, at 28 days after OVX, Number 28 means 28 days after start of administration, at sacrifice. All animals were overnight fasted before OVX, first administration and sacrifice, respectively.

Supplementary Figure S8

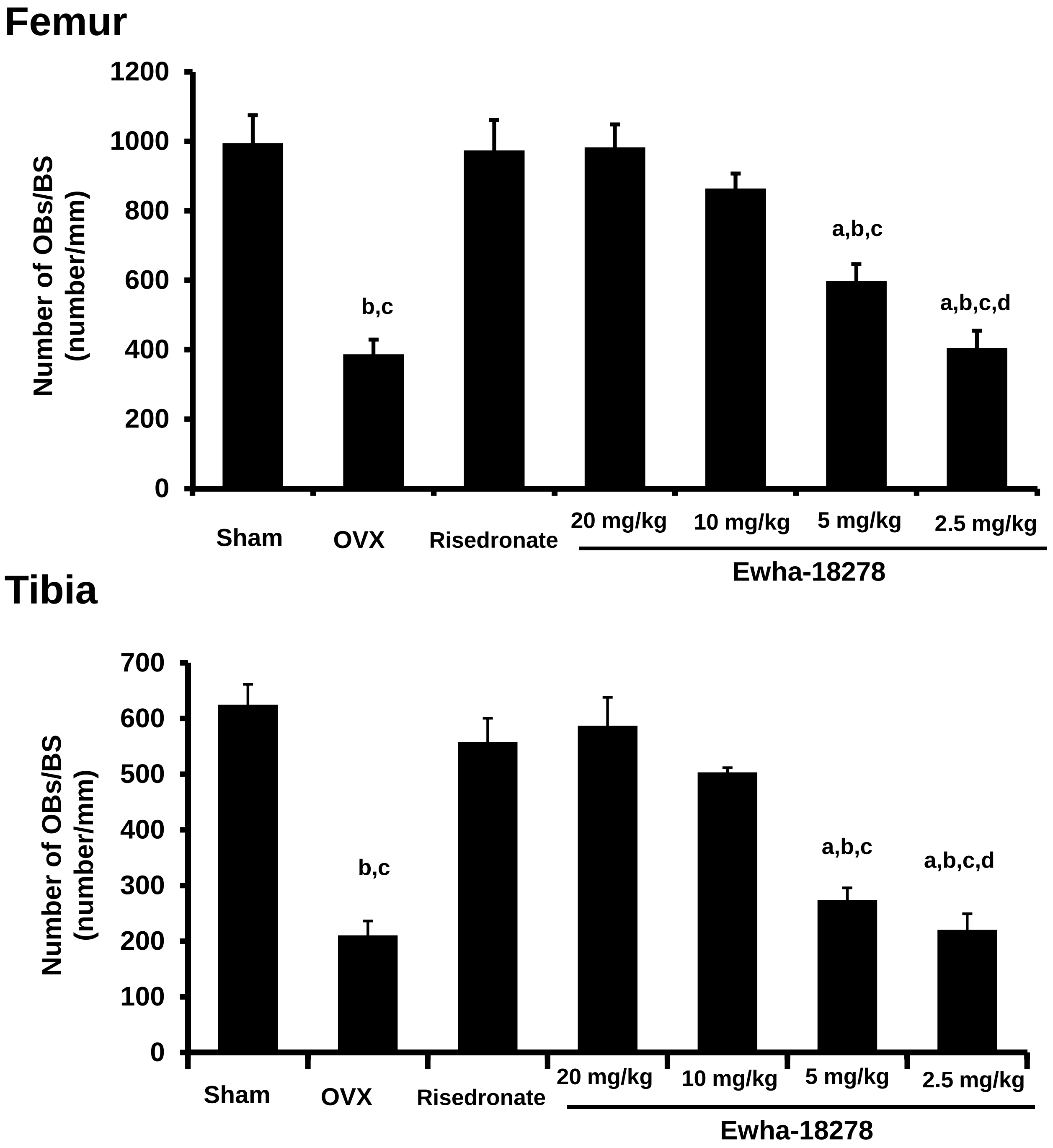

Supplementary Figure S8. Effect of Ewha-18278 on number of OBs in mice. CB number was counted in osteoblastic lining in cortical bone with H&E staining. [a] p<0.005 and [b] p<0.005 as compared with OVX and Risendronate, respectively, [c] p<0.005 and [d] p<0.005 as compared with 20 mg/kg and 10 mg/kg of Ewha-18278, respectively.

Supplementary Table S1

Supplementary Table S1. Pharmacokinetic parameters of Ewha-89403 following IV and oral administration at the doses of 1 mg/kg and 10 mg/kg, respectively, to rats (n=4-5)

PK Parameters	IV Injection	Oral Administration
C_0 (μg/mL)	2.52 ± 0.837	-
C_{max} (μg/mL)	-	0.194 ± 0.054
T_{max} (h)	-	0.102 ± 0.039
$t_{1/2}$ (h)	1.43 ± 0.723	3.30 ± 2.08
V_d (mL)	2643 ± 1410	-
Cl_t (mL/h)	1277 ± 162.1	-
$AUC_{0\rightarrow t}$ (μg·h/mL)	0.343 ± 0.045	0.149 ± 0.039
$AUC_{0\rightarrow \infty}$ (μg·h/mL)	0.418 ± 0.056	0.302 ± 0.133

Absolute Bioavailability (BA) = 4.3 %

Supplementary Table S2

Supplementary Table S2. Pharmacokinetic parameters of Ewha-18278 following IV and oral administration at the doses of 2 mg/kg and 20 mg/kg, respectively, to rats (n=5-6)

PK Parameters	IV Injection	Oral Administration
**C_0 (μg/mL)**	**7.48 ± 3.42**	**-**
**C_{max} (μg/mL)**	**-**	**5.01 ± 2.51**
**T_{max} (h)**	**-**	**0.233 ± 0.171**
**$t_{1/2}$ (h)**	**4.07 ± 2.28**	**14.4 ± 3.73**
**V_d (mL)**	**1523 ± 1170**	**-**
**Cl_t (mL/h)**	**250 ± 72.3**	**-**
**$AUC_{0\rightarrow t}$ (μg·h/mL)**	**1.77 ± 0.697**	**11.3 ± 6.47**
**$AUC_{0\rightarrow\infty}$ (μg·h/mL)**	**2.15 ± 0.693**	**14.4 ± 8.17**

Absolute Bioavailability (BA) = 63.8 %

Supplementary Table S3

Supplementary Table S3. Femur Weights in OVX Mice Values are expressed mean ± S.D. of eight mice. Risedronate sodium was administered at 2.5mg/kg levels [a] $p<0.01$ and [b] $p<0.05$ as compared with sham control by LSD test [c] $p<0.01$ and [d] $p<0.05$ as compared with OVX control by LSD test [e] $p<0.01$ and [f] $p<0.05$ as compared with sham control by MW test [g] $p<0.05$ as compared with OVX control by MW test Ewha-18278 = test material OVX = ovariectomy

Groups	Femur absolute weights (g)			Femur relative weights (% of body weight)		
	Wet	Dry	Ash	Wet	Dry	Ash
**Controls**						
Sham	0.089±0.016	0.070±0.006	0.040±0.002	0.291±0.056	0.229±0.020	0.130±0.007
OVX	0.098±0.011	0.063±0.004[a]	0.032±0.003[a]	0.273±0.028	0.175±0.021[a]	0.089±0.015[e]
Risedronate	0.096±0.002	0.066±0.002[b]	0.037±0.002[d]	0.263±0.012	0.181±0.011[a]	0.102±0.007[e]
**Ewha-18278**						
20.0mg/kg	0.096±0.006	0.067±0.002	0.038±0.004[c]	0.264±0.013	0.183±0.007[a]	0.104±0.007[eg]
10.0mg/kg	0.096±0.008	0.067±0.004[d]	0.038±0.004[c]	0.271±0.024	0.190±0.018[a]	0.108±0.018[fg]
5.0mg/kg	0.093±0.008	0.065±0.005[b]	0.037±0.004[c]	0.267±0.026	0.185±0.019[a]	0.106±0.012[eg]
2.5mg/kg	0.097±0.008	0.065±0.006[b]	0.030±0.007[a]	0.266±0.023	0.180±0.025[a]	0.082±0.019[e]

Supplementary Table S4

Supplementary Table S4. Tibia Weights in OVX Mice Values are expressed mean ± S.D. of eight mice Risedronate sodium was administered at 2.5mg/kg levels [a] p<0.01 as compared with sham control by LSD test [b] p<0.01 and [c] p<0.05 as compared with OVX control by LSD test Ewha-18278 = test material OVX = ovariectomy

Groups	Tibia absolute weights (g)			Tibia relative weights (% of body weight)		
	Wet	Dry	Ash	Wet	Dry	Ash
**Controls**						
Sham	0.073±0.002	0.051±0.003	0.032±0.003	0.238±0.016	0.169±0.012	0.108±0.011
OVX	0.076±0.010	0.045±0.004[a]	0.026±0.003[a]	0.211±0.024[a]	0.126±0.014[a]	0.071±0.008[a]
Risedronate	0.071±0.003	0.050±0.003[b]	0.031±0.002[b]	0.196±0.012[a]	0.137±0.007[ac]	0.085±0.008[ab]
**Ewha-18278**						
20.0mg/kg	0.071±0.004	0.052±0.003[b]	0.032±0.003[b]	0.195±0.008[a]	0.142±0.008[ab]	0.087±0.009[ab]
10.0mg/kg	0.071±0.004	0.052±0.003[b]	0.030±0.003[b]	0.200±0.015[a]	0.146±0.008[ab]	0.084±0.006[ab]
5.0mg/kg	0.071±0.004	0.051±0.003[b]	0.030±0.002[b]	0.202±0.016[a]	0.146±0.011[ab]	0.086±0.007[ab]
2.5mg/kg	0.070±0.006	0.051±0.004[b]	0.027±0.004[a]	0.193±0.020[a]	0.140±0.010[ab]	0.074±0.013[a]
